# CHI3L1 Expression in Neutrophils and Plasma of Multiple Sclerosis Patients: Implications for Pathogenesis and a Potential Biomarker

**DOI:** 10.3390/ijms27052186

**Published:** 2026-02-26

**Authors:** Izabela Jatczak-Pawlik, Alicja Ewiak-Paszyńska, Małgorzata Domowicz, Bartosz Bielecki, Mariola Świderek-Matysiak, Mariusz Stasiołek, Anna Jurewicz

**Affiliations:** Department of Neurology, Medical University of Lodz, Kosciuszki Street 4, 90-419 Lodz, Poland; izabela.jatczak@umed.lodz.pl (I.J.-P.); ewiak.alicja@gmail.com (A.E.-P.); malgorzata.domowicz@umed.lodz.pl (M.D.); bartosz.bielecki@umed.lodz.pl (B.B.); mariola.swiderek-matysiak@umed.lodz.pl (M.Ś.-M.); anna.jurewicz@umed.lodz.pl (A.J.)

**Keywords:** multiple sclerosis, neutrophils, CHI3L1, CD66b, CD16, lactoferrin

## Abstract

This study investigated the expression and subcellular localization of chitinase-3-like protein 1 (CHI3L1) in neutrophils and plasma from untreated and dimethyl fumarate (DMF)-treated multiple sclerosis (MS) patients, and healthy controls. Intracellular CHI3L1 expression was assessed in CD66b+ neutrophils and CD16+ cells using flow cytometry. Subcellular localization was analyzed by confocal microscopy using markers for various neutrophil granules, while ELISA measured plasma CHI3L1 and lactoferrin levels. We found that both intracellular CHI3L1 levels (expressed as mean fluorescence intensity, MFI) and the proportion of CD66b+ cells were significantly increased in MS patients compared to healthy controls. CHI3L1 was found to colocalize with CD66b+ specific granules. While plasma CHI3L1 levels in untreated MS patients remained comparable to those of healthy controls, a significant increase in both intracellular and plasma CHI3L1 was observed in DMF-treated MS patients. The lack of correlation between plasma lactoferrin and CHI3L1 might suggest selective release mechanisms or differential synthesis of these proteins, despite their common storage in specific granules. These findings highlight the role of neutrophils as a peripheral source of CHI3L1 and suggest a complex association between neutrophil-derived CHI3L1 and the differences observed in DMF-treated MS patients.

## 1. Introduction

Multiple sclerosis (MS) is a chronic autoimmune demyelinating disease of the central nervous system (CNS), which is characterized by inflammation, demyelination, axonal damage, astrogliosis, and neurodegeneration. These processes lead to neurological deficits [1]. Although the precise etiology of MS is not fully understood, the obtained data suggests a multifactorial origin including genetic susceptibility, immunopathological mechanisms, and environmental factors such as vitamin D deficiency, smoking, Epstein–Barr virus (EBV) infection, and obesity [2,3]. The disease predominantly affects young adults, with an onset between 20 and 40 years of age, and a marked female predominance (with a female: male ratio of 3:1) [4]. The complex nature of MS and lack of fully specific diagnostic markers often leads to delays in both diagnosis and treatment initiation [5,6]. Although magnetic resonance imaging (MRI) is a crucial diagnostic tool in MS, its results are not always definitive, and it possesses inherent limitations [7,8]. To address these diagnostic and prognostic difficulties, ongoing research focuses on identifying minimally invasive biomarkers detectable in body fluids that should be essential for early diagnosis, prognosis, and monitoring treatment efficacy [9]. Among them, a glycoprotein chitinase 3-like protein 1 (CHI3L1) has emerged as a promising candidate [10,11,12].

This glycoprotein, also known as YKL-40, is a 40 kDa molecule encoded by the *CHI3L1* gene, located on chromosome 1q32.1. Despite belonging to glycosyl hydrolase family 18 (GH18) and to a subgroup of chitinase-like proteins (CLPs), it lacks enzymatic activity but retains high affinity for chitin [13,14]. This suggests that its main role depends on non-enzymatic functions likely mediated through binding to several cell receptors [15]. The glycoprotein plays a complex, multifaceted role in various biological processes, including defense against pathogens, regulation of the injury response, promotion of anti-inflammatory M2 macrophage differentiation, facilitation of tissue repair, and participation in inflammation, apoptosis, and inflammasome activation [16]. It is secreted by various cell types, including synoviocytes, chondrocytes, endothelial cells, neutrophils, and CNS-resident cells such as microglia and astrocytes [17].

Under chronic inflammatory conditions in MS, elevated CHI3L1 levels in the cerebrospinal fluid (CSF) have been shown to correlate with disease progression, brain atrophy, and higher expanded disability status scale (EDSS) scores [18]. Furthermore, high CHI3L1 expression has been associated with pathology in normal-appearing grey matter [19]. In the CNS, its expression is primarily driven by activated astrocytes and microglia [20], where it serves as a biomarker of chronic inflammation and active neurodegeneration [21]. Conversely, elevated serum CHI3L1 levels in MS patients [12,22,23] have been attributed to its secretion by peripheral immune cells—predominantly neutrophils [24]–as well as endothelial cells [25] and smooth muscle cells [26].

While CHI3L1 is not specific to MS, as it is elevated in a wide array of systemic disorders [27,28,29,30,31], its levels often correlate with the degree of inflammation, allowing for the assessment of disease progression and treatment response. Dimethyl fumarate (DMF) is an established disease-modifying therapy in MS, known for its immunomodulatory and neuroprotective effects, primarily mediated through the activation of the nuclear factor erythroid 2-related factor 2 (Nrf2) antioxidant pathway [32]. Beyond its antioxidant properties, DMF significantly influences the peripheral immune system by attenuating the activity of pro-inflammatory cells and modulating neutrophil functions [33,34]. However, its specific impact on CHI3L1 synthesis and secretion patterns remains unclear.

Our study aimed to provide novel insights into CHI3L1 expression in MS by comparing untreated and DMF-treated patients with healthy controls. We focused on investigating the intracellular expression and subcellular localization of CHI3L1 within neutrophil populations, as well as evaluating its levels in plasma. Furthermore, we intended to determine whether CHI3L1 release patterns correlate with established markers of degranulation, such as lactoferrin. By analyzing these parameters, we aimed to clarify the potential role of neutrophils in MS pathogenesis and to identify differences in CHI3L1 profiles associated with DMF treatment.

## 2. Results

The demographic and clinical characteristics of the MS patients and healthy controls are summarized in Table 1.

### 2.1. Increased Intracellular CHI3L1 Expression in CD66b+ Neutrophils and CD16+ Cells of MS Patients

#### 2.1.1. Flow Cytometry Results

**Figure 1:**
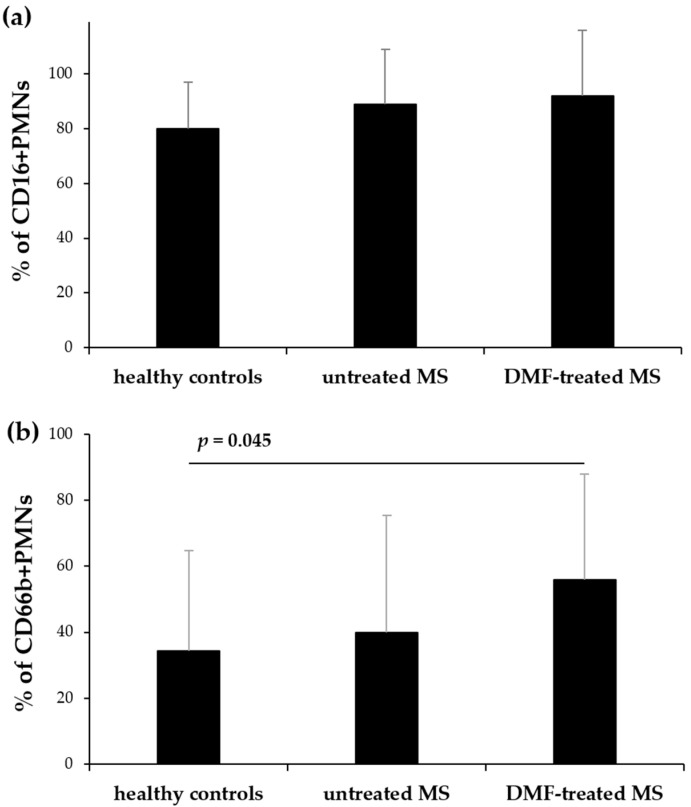
Flow cytometric analysis of CD16 (**a**) and CD66b (**b**) expression on PMNs isolated from healthy controls, untreated, and DMF-treated MS patients. The PMN population was defined as 100% for this analysis. Data are presented as mean ± SD. Statistical significance was determined using one-way ANOVA followed by Tukey’s post hoc test, *p* < 0.05.

**Figure 2:**
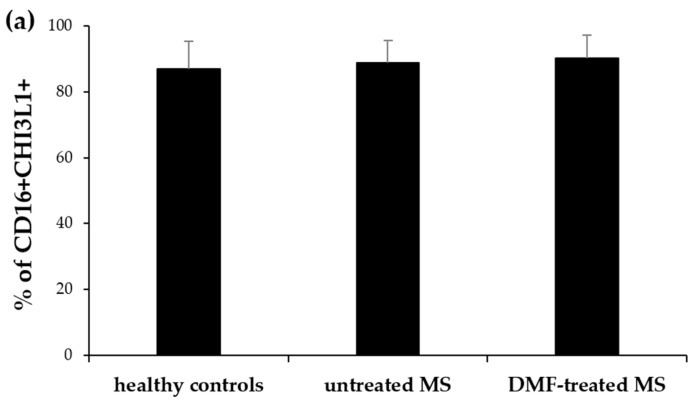

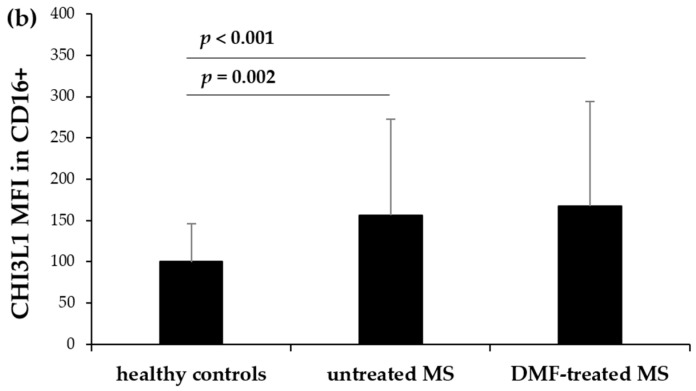
Flow cytometric analysis of CHI3L1 expression in CD16+ cells isolated from healthy controls, untreated, and DMF-treated MS patients. The CD16+ cell population was defined as 100% for this analysis. The mean percentage of CD16+ cells that express CHI3L1 (**a**) and the mean MFI value of CHI3L1 within these cells (**b**). The data are presented as the mean ± SD. Statistical significance was determined using one-way ANOVA followed by Tukey’s post hoc test, *p* < 0.05.

**Figure 3:**
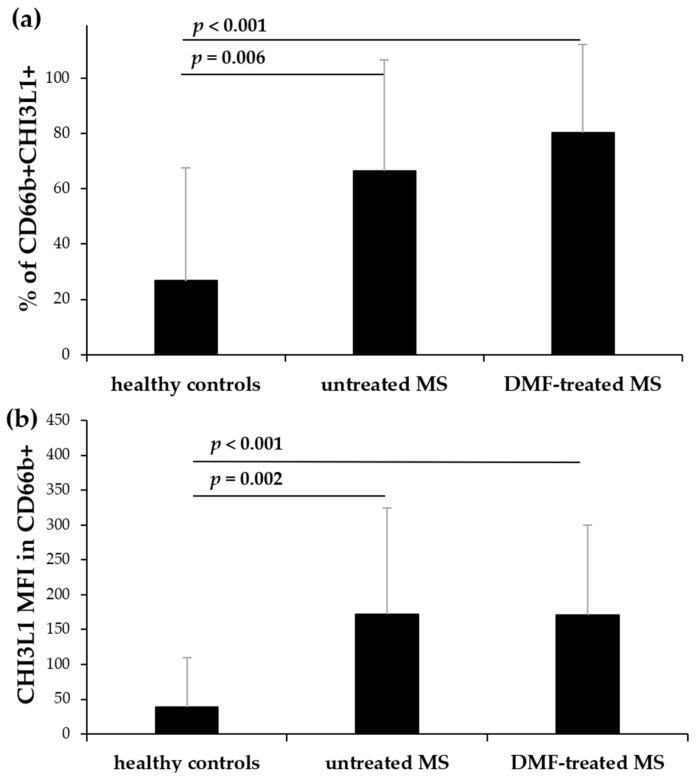
Flow cytometric analysis of CHI3L1 expression in CD66b+ cells isolated from healthy controls, untreated, and DMF-treated MS patients. The CD66b+ cell population was defined as 100% for this analysis. The mean percentage of CD66b+ cells that express CHI3L1 (**a**) and the mean MFI value of CHI3L1 within these cells (**b**). The data are presented as the mean ± SD. Statistical significance was determined using one-way ANOVA followed by Tukey’s post hoc test, *p* < 0.05.

Using flow cytometry, we assessed CHI3L1 expression in neutrophils (CD66b+) and CD16+ cells within polymorphonuclear leukocytes (PMNs) isolated from untreated and DMF-treated MS patients, and healthy controls. The proportion of CD16+ cells remained comparable among all groups (Figure 1a). However, the proportion of CD66b+ cells was significantly higher in DMF-treated patients than in healthy controls (*p* = 0.045) (Figure 1b). While no significant differences were observed in the percentage of CD16+ cells expressing intracellular CHI3L1 among the groups (Figure 2a), the proportion of CD66b+ cells expressing CHI3L1 was significantly higher in both untreated (*p* = 0.006) and DMF-treated MS patients (*p* < 0.001) in comparison to healthy controls (Figure 3a).

**Figure 4:**
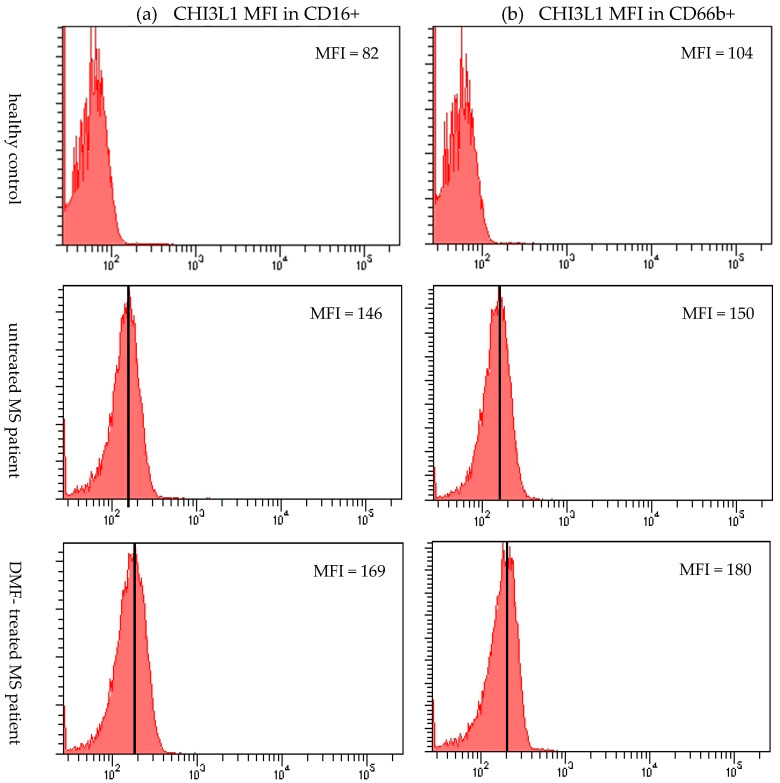
Representative histograms of the intracellular expression of CHI3L1 in CD16+ cells (**a**) and CD66b+ (neutrophils) (**b**) isolated from a healthy control, an untreated MS patient, and a DMF-treated MS patient. The arithmetic mean MFI value of CHI3L1 for each cell population is indicated next to its corresponding histogram.

The mean fluorescence intensity (MFI) of CHI3L1 in CD16+ cells was markedly higher in both untreated (*p* = 0.002) and DMF-treated MS patients (*p* < 0.001) compared to healthy controls (Figure 2b and Figure 4a). Similarly, CHI3L1 MFI in CD66b+ cells was significantly elevated in both MS patient groups (*p* = 0.002 and *p* < 0.001, respectively) in comparison to healthy controls (Figure 3b and Figure 4b). Detailed values for CHI3L1 expression and neutrophil subpopulation characteristics, including means and standard deviations (SD), are summarized in Table 2 (see Section 2.3). In line with previous reports [16,35,36], CHI3L1 was not detected in CD14+ monocytes or CD3+ T lymphocytes.

#### 2.1.2. Immunofluorescence Staining Results

**Figure 5:**
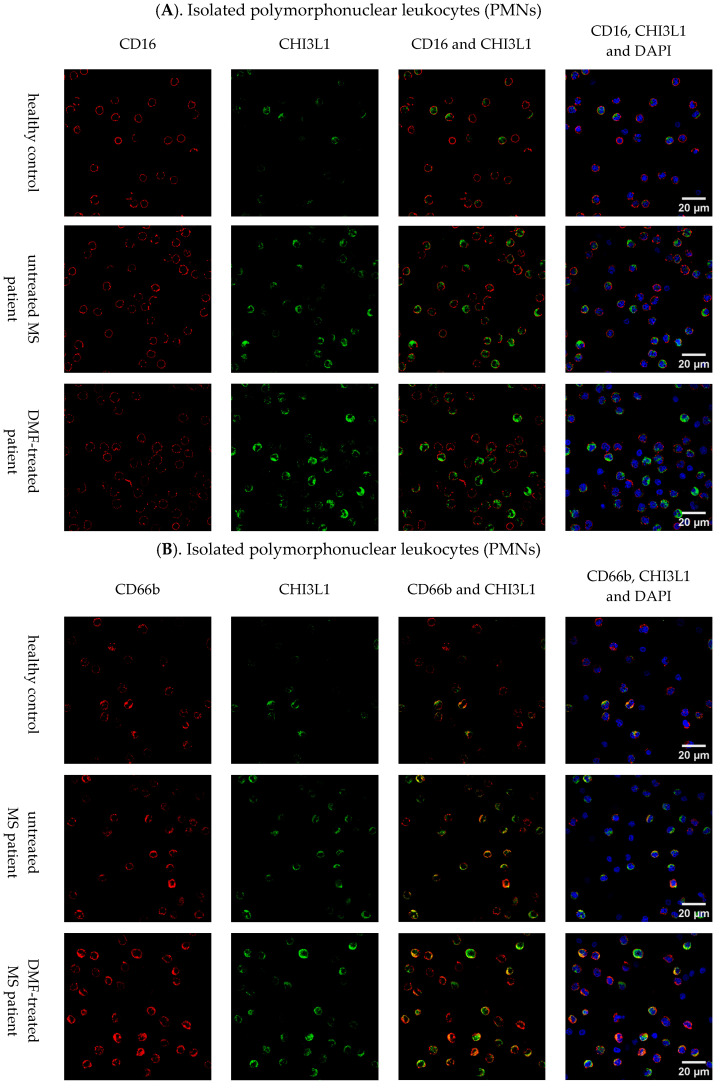

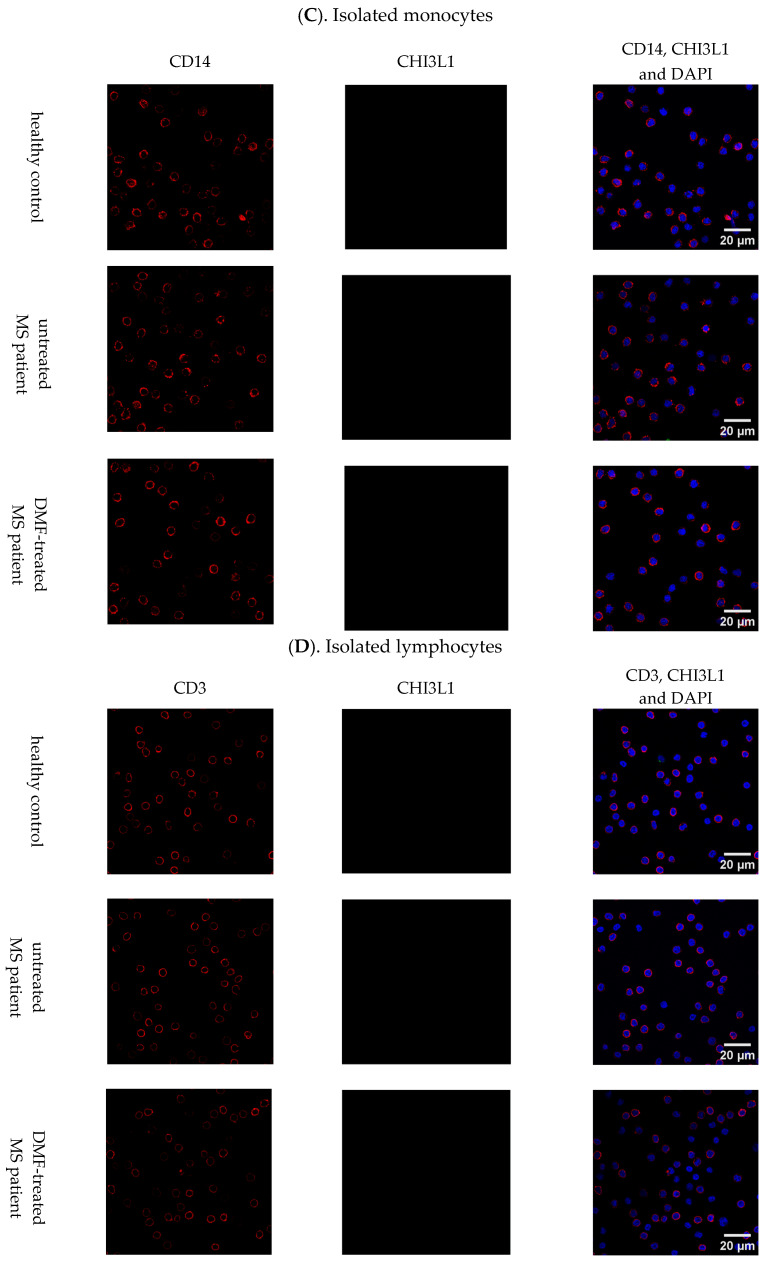
Representative images of CHI3L1 expression in leukocyte subsets. CHI3L1 (green) was assessed in CD16+ (**A**) and CD66b+ (**B**) PMNs, monocytes (CD14+) (**C**), and T lymphocytes (CD3+) (**D**) isolated from healthy controls, untreated, and DMF-treated MS patients. Cell-specific markers (CD16, CD66b, CD14, and CD3) are shown in red, and nuclei were counterstained with DAPI (blue). The images were acquired with an Olympus Fluoview FV3000 confocal laser scanning microscope (Evident/Olympus, Tokyo, Japan) and are representative of five independent experiments. Scale bars = 20 µm apply to all panels in the row.

To further validate our flow cytometry findings, CHI3L1 expression was assessed by confocal microscopy across the leukocyte populations, yielding results consistent with the quantitative flow cytometric data. In Figure 5A, while the number of CD16+ cells remained similar across all groups, those from MS patients (untreated and DMF-treated) displayed a noticeably stronger and more distinct signal for CHI3L1 compared to healthy controls. Subsequently, as shown in Figure 5B, a higher number of CD66b+ neutrophils expressing CHI3L1 was observed in both untreated and DMF-treated MS patients. Merged images of CD66b (red) and CHI3L1 (green) revealed a consistent orange-yellow granular pattern, providing direct visual evidence of the colocalization of these proteins. While this colocalization was a constant biological feature observed in all groups, the signal was more abundant in samples from MS patients, reflecting the expansion of the CHI3L1-positive neutrophil population.

In line with the flow cytometry data, no CHI3L1 expression was detected in CD14+ monocytes (Figure 5C) or CD3+ T lymphocytes (Figure 5D) from either MS patients or healthy controls.

### 2.2. CHI3L1 Is Localized Within the Specific Granules of Neutrophils

**Figure 6:**
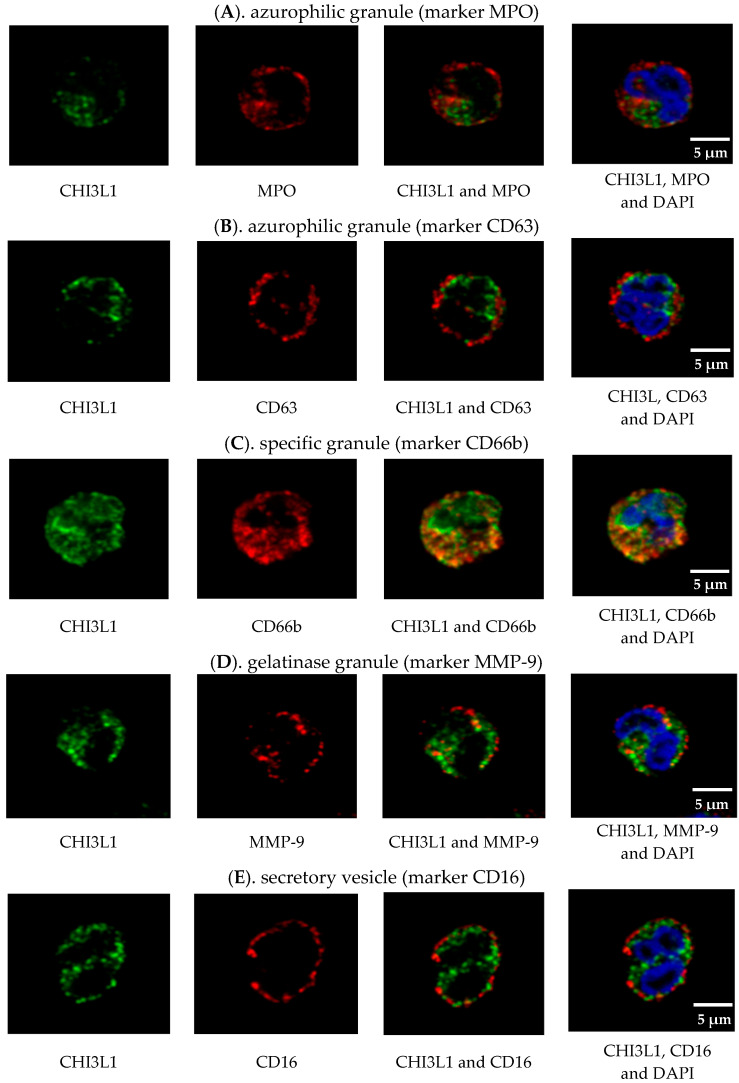
CHI3L1 (green) colocalizes with markers of neutrophil granules and vesicles in MS patients. Representative confocal images show CHI3L1 expression alongside MPO (**A**), CD63 (**B**), CD66b (**C**), MMP-9 (**D**), and CD16 (**E**) (all in red). The nuclei were counterstained with DAPI (blue). The images were acquired using a 60× oil immersion objective (Olympus Fluoview FV3000). High-magnification crops are shown to visualize subcellular details. Scale bars = 5 µm apply to all panels in the row. Data are representative of three independent experiments.

To investigate the subcellular localization of CHI3L1 in neutrophils isolated from untreated MS patients, we analyzed the colocalization of CHI3L1 with markers for various granules and secretory vesicles. These markers included myeloperoxidase (MPO) and CD63 specific for azurophil granules; matrix metalloproteinase-9 (MMP-9) for gelatinase granules; CD66b for specific granules; and CD16 for secretory vesicles. Analysis revealed a strong positive correlation between CHI3L1 and CD66b (Pearson’s coefficient *r* = 0.66 ± 0.07), visually manifested as a consistent orange-yellow granular pattern in merged images (Figure 6C). Furthermore, significant overlap was demonstrated by the thresholded Manders’ *M*_1_ value (0.76 ± 0.08), indicating that 76% of the CD66b signal was found in the areas containing CHI3L1; meanwhile, the *M*_2_ value (0.36 ± 0.05) indicates that 36% of the CHI3L1 signal is located within the areas containing CD66b. No colocalization was observed with other markers, including MPO, CD63, MMP-9, and CD16 (Figure 6A,B,D,E). These observations were confirmed by low values of Pearson’s coefficient (ranging from 0.17 to 0.30) and very low Manders’ coefficients (*M*_2_ ranging from 0.01 to 0.03). These findings strongly suggest that CHI3L1 is predominantly located in CD66b-containing specific granules (Figure 6C). Since CD66b is an integral membrane protein expressed on both external and intracellular membranes, the observed Manders’ coefficients can be explained by the restricted colocalization of CHI3L1 inside the granules, whereas CD66b is also present on the cell surface [24,37].

### 2.3. Increased CHI3L1 but Not Lactoferrin Plasma Concentrations in MS Patients

**Figure 7:**
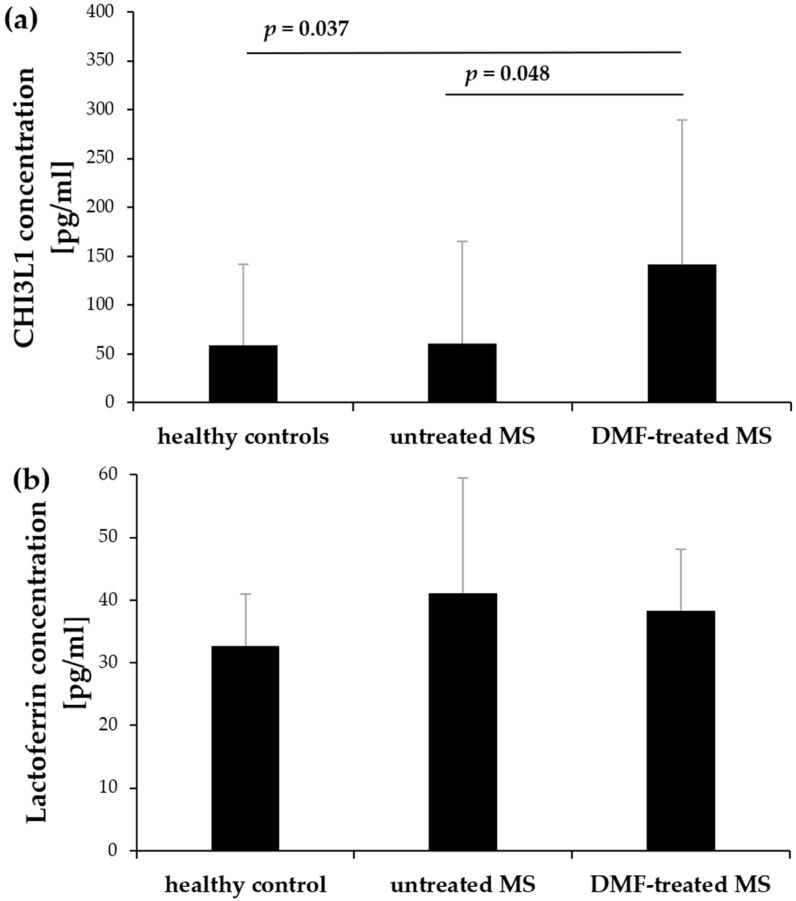
CHI3L1 (**a**) and lactoferrin (**b**) plasma concentrations in healthy controls, untreated, and DMF-treated MS patients were assessed using ELISA. Data are presented as mean ± SD. Statistical significance was determined using one-way ANOVA followed by Tukey’s post hoc test, *p* < 0.05.

The concentrations of CHI3L1 and lactoferrin were measured in plasma samples obtained from healthy controls, untreated, and DMF-treated MS patients using enzyme-linked immunosorbent assays (ELISA). The CHI3L1 concentration was significantly higher in DMF-treated patients compared to both healthy controls (*p* = 0.037) and untreated MS patients (*p* = 0.048). However, CHI3L1 plasma concentrations in untreated MS patients did not differ significantly from those of healthy controls (Figure 7a). Furthermore, no significant differences in lactoferrin plasma concentrations were observed among studied groups (Figure 7b). The exact mean and SD values for these plasma concentrations are summarized in Table 2.

**Table 2:** Mean value and SD of neutrophil characteristics (flow cytometry) and plasma protein concentrations (ELISA).

Parameters	Healthy Controls	Untreated MS Patients	DMF-Treated MS Patients
cell surface marker (flow cytometry)			
% of CD16+ PMNs	80.5 ± 17.5	89.2 ± 19.1	92.4 ± 22.5
% of CD66b+ PMNs	34.4 ± 30.7	40.0 ± 35.5	56.2 ± 33.4
intracellular expression (flow cytometry)			
% of CD16+ CHI3L1+	87.2 ± 8.1	89.2 ± 7.5	90.5 ± 7.4
CHI3L1 MFI in CD16+	101.3 ± 45.1	158.2± 114.0	168.7 ± 127.5
% of CD66b+ CHI3L1+	26.8 ± 40.8	66.5 ± 40.5	80.5 ± 33.1
CHI3L1 MFI in CD66b+	40.3 ± 70.1	172.5 ± 152.4	171.8 ± 128.8
secreted protein (ELISA)			
CHI3L1 [pg/mL]	58.5 ± 83.2	60.9 ± 105.2	141.5 ± 148.1
lactoferrin [pg/mL]	32.7 ± 8.5	41.1 ± 18.3	38.4 ± 9.7

**Notes:** Data derived from flow cytometric and ELISA analyses. Statistical significance was determined using one-way ANOVA followed by Tukey’s post hoc test. **Abbreviations:** CHI3L1, chitinase-3-like protein 1; DMF, dimethyl fumarate; ELISA, enzyme-linked immunosorbent assay; MFI, mean fluorescence intensity; MS, multiple sclerosis; PMNs, polymorphonuclear leukocytes.

### 2.4. Correlation Between Neutrophil CHI3L1 Expression and Disability Status in MS Patients

**Figure 8:**
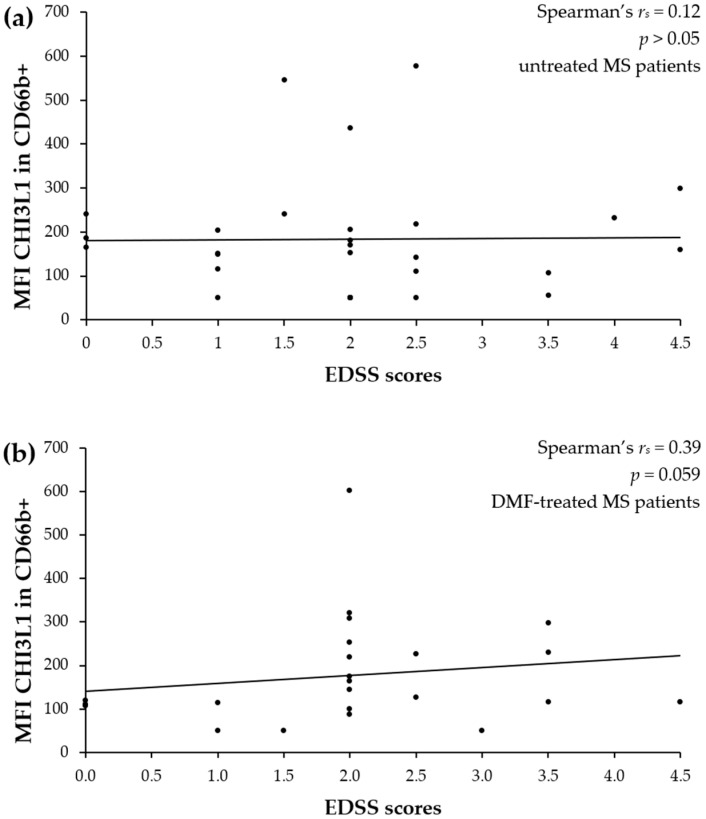
Correlation between intracellular CHI3L1 MFI in CD66b+ neutrophils and EDSS scores in untreated MS patients (**a**) and DMF-treated MS patients (**b**). Analysis was performed using Spearman’s rank correlation coefficient. The solid line represents the linear regression trend.

To determine whether elevated intracellular CHI3L1 expression reflects the clinical severity of the disease, a correlation analysis was performed between CD66b+ neutrophil CHI3L1 MFI and EDSS scores in MS patients. No significant correlation was observed for untreated MS patients (Spearman’s *r_s_* = 0.12, *p* > 0.05; Figure 8a). In the DMF-treated group, although the correlation did not reach statistical significance, a weak-to-moderate positive trend was observed (Spearman’s *r_s_* = 0.39, *p* = 0.059; Figure 8b).

## 3. Discussion

Our study provides significant insights into CHI3L1 expression within the neutrophils of MS patients, examining both untreated individuals and those receiving DMF therapy. It is important to emphasize that the untreated relapsing-remitting MS (RRMS) group included mainly patients at the onset of the disease, with a yet to be determined natural disease progression. Furthermore, it must be explicitly acknowledged that the cross-sectional design of this study means the longer disease duration in the DMF-treated group is inherently linked to the observed treatment effects. Previous studies have consistently demonstrated elevated CHI3L1 levels in the CSF and serum of MS patients, particularly in the progressive forms of MS, compared to RRMS and healthy controls [18,23,38]. Increased CHI3L1 levels often correlate with disability progression, as measured by the EDSS score and pronounced brain atrophy, suggesting its potential role as a valuable biomarker for disease monitoring [18,38]. Furthermore, high CHI3L1 levels in patients with clinically isolated syndrome (CIS) correlate with faster conversion to clinically definite MS [22,39]. While reactive astrocytes and macrophages/microglia are well-established CNS sources of CHI3L1 [22,40], the origin of peripheral CHI3L1 is less defined. Increasing evidence suggests that neutrophils could be a major source of systemic CHI3L1 [22,39,40]. Consequently, we focused our study on CD66b+ neutrophils and CD16+ cells. The CD66b molecule, a marker of specific granules and degranulation, was used to define neutrophil activation status [41]. CD16 serves as a marker for mature neutrophils, despite its limited expression on natural killer (NK) cells and certain monocyte subsets (e.g., CD14+CD16+ monocytes) [42]. To confirm the specificity of the CD16 marker, the CD14+CD16+ monocyte population was assessed and represented less than 1.8% of the total CD16+ cells across all groups.

Our analysis revealed that while the total mature granulocyte population (defined as CD16+, PMNs, Figure 1a) remained unchanged among groups, the intracellular expression of CHI3L1 per cell was significantly higher in MS patients. This was evidenced by the significantly elevated MFI of CHI3L1 in CD16+ cells (Figure 2b and Figure 4a). In contrast to the consistent proportion of CD16+ cells, the percentage of CD66b+ cells in PMNs was significantly increased in DMF-treated MS patients compared to healthy controls (Figure 1b). Parallel to our findings in CD16+ cells, a significantly higher MFI of CHI3L1 was also observed within the CD66b+ population in both the MS group (Figure 3b and Figure 4b). Furthermore, although the percentage of CHI3L1-expressing CD16+ cells remained stable (Figure 2a), the proportion of CHI3L1-expressing CD66b+ cells was significantly increased in both MS groups (Figure 3a). These elevations, observed in untreated patients at the clinical onset of RRMS, suggest that neutrophil activation is a remarkably early event in MS pathogenesis. This indicates that CHI3L1-associated inflammation is not merely a consequence of chronic disease or cumulative treatment, but rather a sensitive indicator of early systemic immune priming that precedes substantial axonal damage and clinical disability.

The observed upregulation of CD66b, which was the most pronounced in the DMF group, likely reflects a state of enhanced systemic immune priming. Since the total CD16+ population remained unchanged (Figure 1a), the increased proportion of CD66b+ cells (Figure 1b) suggests enhanced neutrophil activation in DMF-treated MS patients. These findings suggest that while DMF limits CNS infiltration [43], it might not necessarily suppress the initial synthesis of inflammatory mediators, such as CHI3L1, within the bone marrow or peripheral blood compartments.

Confocal microscopy confirmed that CHI3L1 was colocalized exclusively with CD66b, indicating its storage within specific granules, but was not detected in azurophilic granules (MPO/CD63), gelatinase granules (MMP-9), and secretory vesicles (CD16) (Figure 6). Interestingly, the elevated intracellular CHI3L1 expression in neutrophils of MS patients contrasted with the less pronounced differences in plasma concentrations between untreated patients and healthy controls (Figure 7a). This discrepancy might be explained by the fact that resting neutrophils entering the bloodstream undergo only a priming process. This state–triggered by agents such as tumor necrosis factor alpha (TNFα), IL-6, or IL-8–does not result in full degranulation [44]. The critical signal for the release of these granular proteins is the adhesion of neutrophils to the inflamed vascular endothelium, a process mediated by *β*_2_ (CD18) integrins [45]. The requirement of adhesion-derived signal for degranulation suggests that CHI3L1 might accumulate within neutrophils until their migrate across the blood–brain barrier (BBB). This idea might be supported by the detection of neutrophils in the CSF of MS patients [46] and the demonstration of the neutrophil capacity to migrate across the BBB in an animal model of MS [47]. Therefore, the neutrophils might serve as a source of CHI3L1 localized within the CNS rather than a systemic one. This hypothesis is further supported by our data on CHI3L1 production and secretion during HL-60 human leukemia cell line differentiation and maturation [48].

To assess the possibility of in vivo CHI3L1 release via degranulation, we compared plasma levels of CHI3L1 with lactoferrin, another established marker of specific granules [37]. Since both proteins are stored within the same granular compartment, they would be expected to be co-released simultaneously during neutrophil activation. However, the increased plasma CHI3L1 levels in DMF-treated patients did not correlate with lactoferrin concentrations (Figure 7a,b), suggesting distinct regulatory pathways. This dissociation might indicate selective secretion of CHI3L1 via neutrophil extracellular traps (NETs), a process characterized by the release of granule contents independent of classical degranulation, which is known to be influenced by DMF [33,34]. Alternatively, this discrepancy could result from differential synthesis rates [10,49] or post-translational modifications affecting the serum stability of these proteins [50,51].

Consistent with previous research showing that DMF interferes with neutrophil adhesion and chemotaxis, thereby reducing CNS infiltration [43], we propose a “peripheral trapping” hypothesis. While DMF does not deplete the total neutrophil count (Figure 1a, [43,52]), it appears to impede their migration across the BBB. Consequently, these “trapped”, activated, CHI3L1-rich neutrophils likely accumulate in the systemic circulation, potentially releasing CHI3L1 into the plasma (Figure 7a) instead of the CNS compartment. Crucially, these systemic inflammatory changes appear to be decoupled from the patients’ current neurological disability. The lack of correlation between intracellular CHI3L1 levels and EDSS scores in both groups (Figure 8) suggests that while CHI3L1 reflects active, ongoing biochemical inflammation, EDSS scores primarily reflect accumulated past axonal damage. While CHI3L1 might also exert neuroprotective effects by promoting an anti-inflammatory M2 microglial phenotype [53,54], further longitudinal studies are required to determine the long-term clinical implications of this peripheral retention.

From a clinical perspective, monitoring neutrophil CHI3L1 levels might offer valuable insights into a patient’s inflammatory status. Specifically, parameters such as CHI3L1 MFI in neutrophils could serve as biomarkers to guide treatment adjustments and support personalized MS management. By identifying subtle variations in therapeutic responses, these markers could facilitate more evidence-based decisions regarding treatment optimization and long-term monitoring.

Certain limitations of this study warrant consideration. First, as noted, the cross-sectional nature of our study design prevents a definitive separation of treatment effects in the DMF-treated group from the influence of disease duration. Second, the untreated MS group represents a clinically heterogeneous population at disease onset. This heterogeneity may have increased within-group variance, potentially masking or, conversely, inflating differences observed between the study groups. Furthermore, it is important to acknowledge that the present study did not evaluate the biomechanical properties of neutrophils, such as cell stiffness, shape, or adhesion dynamics. These physical characteristics are essential for understanding how neutrophils migrate across the BBB [55]. While our results focus on the biochemical role of CHI3L1, the absence of biomechanical data represents a further limitation that should be addressed in future studies to provide a more comprehensive view of neutrophil behavior in MS.

In conclusion, our study identifies circulating neutrophils as a significant source of CHI3L1 in MS, acting as a sensitive early indicator of inflammation that precedes permanent physical disability. The subcellular localization of CHI3L1 within specific granules, together with the observed dissociation from lactoferrin in DMF-treated patients, highlights complex, protein-specific release mechanisms, potentially involving selective NETosis. These findings underscore the importance of further investigation into the role of neutrophils in MS pathogenesis and the impact of treatment-associated differences on inflammatory biomarker profiles.

## 4. Materials and Methods

### 4.1. Patients

There were three study groups: untreated patients with MS, DMF-treated MS patients, and healthy controls (Table 1, see Section 2). The untreated MS patients were newly diagnosed with clinically definite MS according to the 2017 McDonald criteria [56]. These patients were free from clinical activity and steroid treatment for at least 3 months before blood sampling. A baseline brain MRI, performed within 3 months before blood collection, showed no gadolinium-enhancing lesions. DMF-treated MS patients received DMF for a mean duration of 4 ± 2 years, without relapses, and had initiated therapy shortly after diagnosis. These patients were clinically and radiologically stable on their first disease-modifying drug (DMD). Their brain MRI was also performed within 3 months before blood sampling. The participants were recruited from patients of the Department of Neurology, Barlicki Hospital, Medical University of Lodz (Lodz, Poland) between March 2023 and July 2024. The control group consisted of healthy individuals who volunteered as participants.

This study was conducted in accordance with the ethical principles outlined in the Declaration of Helsinki and approved by the Local Ethics Committee of the Medical University of Lodz, Poland (RNN/214/22/KE, 13 September 2022). Written informed consents were obtained from all study participants before any further procedures.

### 4.2. Study Design and Sample Processing

Peripheral blood (25 mL) was collected from each participant, including MS patients, DMF-treated MS patients, and healthy controls, into tubes containing heparin. The collected blood was used for the isolation of all leukocytes (5 mL), PMNs (5 mL), CD14+ monocytes, and enrichment of lymphocytes (10 mL) and plasma (5 mL). Immediately following collection, samples were processed to isolate specific leukocyte populations and plasma.

#### 4.2.1. Isolation of Leukocytes

To obtain the peripheral leukocyte suspension for flow cytometry, 5 mL of peripheral blood was first subjected to erythrocyte lysis. The blood was gently mixed using a 1:9 dilution in 1× red blood cell (RBC) lysis buffer (150 mM NH_4_Cl, 10 mM KHCO_3_, 0.1 mM EDTA, pH 7.4) and incubated on an orbital shaker at room temperature (RT) until visual confirmation of complete erythrocyte lysis (typically 10 min). The obtained leukocyte suspension was then washed twice with sterile phosphate-buffered saline (PBS, pH 7.4). The washing step included centrifugation at 300× *g* for 10 min at RT. Finally, the leukocyte pellet was resuspended in an appropriate volume of PBS for subsequent flow cytometry.

#### 4.2.2. Isolation of PMNs

PMNs were isolated from 5 mL of peripheral blood using density gradient centrifugation on Polymorphprep^TM^ (Serumwerk, Bernburg, Germany), according to the manufacturer’s protocol. Following isolation, the cells were washed twice with sterile PBS (pH 7.4) by centrifugation at 300× *g* for 10 min at RT. The obtained PMN pellet was resuspended in an appropriate volume of PBS for subsequent immunofluorescence staining.

#### 4.2.3. Isolation of CD14+ Monocytes and Enrichment of Lymphocytes

Peripheral blood (10 mL) was diluted 1:2 with sterile PBS (pH 7.4). Peripheral blood mononuclear cells (PBMCs) were isolated using Histopaque 1077 (Merck, Darmstadt, Germany) density gradient centrifugation according to the manufacturer’s protocol. Following isolation, PBMCs were washed twice with PBS (pH 7.4) by centrifugation at 300× *g* for 10 min at RT. Subsequently, CD14+ monocytes were purified from the PBMC population using the Pan Monocyte Isolation Kit, human (Miltenyi Biotec, Bergisch Gladbach, Germany). This magnetic separation yielded two distinct fractions: purified CD14+ monocytes (collected in the flow-through) and a non-monocyte population predominantly composed of CD3+ T lymphocytes (retained on the column). Both isolated cell populations were washed and resuspended in PBS for subsequent immunofluorescence staining.

#### 4.2.4. Isolation of Plasma

Peripheral blood (5 mL) collected in heparinized tubes was centrifuged at 1500× *g* for 30 min at RT to separate the plasma. The supernatant (plasma) was carefully collected, aliquoted into sterile cryovials, and immediately stored at −80 °C until analysis of CHI3L1 and lactoferrin concentrations by ELISA.

### 4.3. Flow Cytometric Analysis

#### 4.3.1. Antibodies

The antibodies, including mouse monoclonal anti-CD45-APC/Cy7 (clone 2D1), anti-CD16-APC (clone 3G8), anti-CD66b-APC (clone G10F5), anti-CD3-FITC (clone HIT3a), and anti-CD14 FITC (clone M5E2), were purchased from BD Biosciences (San Jose, CA, USA). Rabbit polyclonal anti-CHI3L1-PE was obtained from Bioss Antibodies (Boston, MA, USA).

#### 4.3.2. Staining Procedures

The isolated all leukocytes, by erythrocytes lysis (2 × 10^6^ cells) were washed twice with PBS, resuspended in 50 µL of blocking buffer (4% fetal bovine serum and 2% human serum IgG from Sigma-Aldrich in PBS), and incubated with the anti-cell-surface monoclonal antibodies (anti-CD45, anti-CD3, anti-CD14, anti-CD16, anti-CD66b) for 30 min at 4 °C, using the manufacturer’s recommended concentration. After washing with PBS, the cells were fixed with 2% paraformaldehyde solution (200 µL) for 10 min at 37 °C, followed by permeabilization with 70% ethanol (200 µL) on ice for 30 min. Permeabilized cells were then resuspended in 50 µL of blocking buffer and incubated for 30 min on ice before intracellular staining. Subsequently, the cells were incubated with the anti-CHI3L1-PE antibody at the experimentally determined concentration of 0.25 µg/mL for 20 min on ice and finally resuspended in 0.5 mL of PBS. Light protection was maintained throughout all steps of the staining process. The samples were analyzed by flow cytometry (BD LSR II flow cytometer, BD Biosciences, Franklin Lakes, NJ, USA).

### 4.4. Immunofluorescence Staining

#### 4.4.1. Antibodies

The primary antibodies, including mouse monoclonal anti-CD16 (clone 1001049) and goat polyclonal anti-CHI3L1, were purchased from R&D Systems (Minneapolis, MN, USA). Rabbit monoclonal anti-CD66b (clone BLR111H), rabbit polyclonal anti-CD14, and mouse monoclonal anti-CD3 (clone 13) were obtained from Novus Biologicals (Centennial, CO, USA). A mouse monoclonal anti-MPO antibody (UniProt ID P05164) was from Biorbyt (Cambridge, Cambridgeshire, UK). Mouse monoclonal anti-CD63 (clone MEM-259), monoclonal anti-MMP-9 antibody (clone 5G3), and the polyclonal secondary antibodies: Alexa Fluor 488 conjugated donkey anti-mouse IgG, Alexa Fluor 568 donkey anti-rabbit IgG, and Alexa Fluor 647 donkey anti-goat IgG were all obtained from Thermo Fisher Scientific (Waltham, MA, USA).

#### 4.4.2. Staining Procedure

PMNs, CD14+ monocytes, and CD3+ T lymphocytes were isolated from the peripheral blood of untreated MS patients, DMF-treated MS patients, and healthy controls. The CD14+ monocytes were purified from PBMCs, yielding a non-monocyte fraction predominantly composed of CD3+ T cells. The isolated cell fractions were then resuspended in Dulbecco’s phosphate-buffered saline (DPBS) without calcium and magnesium (Thermo Fisher Scientific, Waltham, MA, USA). They were then seeded at 70,000 cells/well on polysine microscope adhesion slides (Epredia Netherlands B.V., Breda, Netherlands) within wells drawn with an advanced PAP pen (Sigma-Aldrich, St. Louis, MO, USA). Cells were settled and adhered for 1 h at RT in a wet chamber. Samples were fixed with cold 4% paraformaldehyde in PBS for 10 min at RT. Following fixation, cells were washed three times for 5 min with 2% bovine serum albumin (BSA) in DPBS. This was followed by a blocking and permeabilization step using 2% normal donkey serum (NDS)/0.01% Triton X-100 (both from Sigma-Aldrich, St. Louis, MO, USA) in DPBS for 1 h at RT. To analyze CHI3L1 expression in granulocytic populations and identify its intracellular location, granulocytes were incubated with primary antibodies at the following dilutions: CHI3L1 (1:110), CD16 (1:62), CD66b (1:100), MPO (1:100), MMP-9 (1:600), CD63 (1:200). Magnetically sorted CD14+ monocytes were incubated with primary antibodies for CD14 (1:100) and CHI3L1 (1:110). The non-monocyte population was stained for CD3+ T lymphocytes (1:100) and CHI3L1 (1:110). All primary antibody cocktails were prepared in antibody dilution buffer (2% NDS in DPBS) and incubated overnight at 4 °C. Cells were subsequently washed three times for 5 min with DPBS and incubated with secondary antibodies (1:1000) in 2% BSA in DPBS at RT for 60 min, protected from light. After three washes for 5 min with DPBS, samples were mounted with Fluoroshield with DAPI (4′, 6-diamidino-2-phenylindole) histology mounting medium (Sigma-Aldrich, St. Louis, MO, USA). Immunostaining images were obtained using an Olympus Fluoview FV3000 confocal laser scanning microscope equipped with a 60× oil immersion objective. All images were initially captured at 1024 × 1024 px resolution with an optical zoom of 1.6 or 1.9. To highlight subcellular details, representative areas were cropped: for Figure 5, images were cropped to 600 × 600 px, while for Figure 6, high-magnification crops were selected to visualize specific subcellular localization. The acquired images were analyzed with ImageJ 1.54p (National Institutes of Health, Bethesda, MD, USA).

### 4.5. CHI3L1 and Lactoferrin Secretion Assay

Human CHI3L1 and lactoferrin concentrations were measured in plasma samples from MS patients (untreated, *n* = 29; and DMF-treated, *n* = 23) and the healthy controls (*n* = 21) by a commercially available ELISA kit from Wuhan EIAab Science (Wuhan, China). Assays were performed according to the manufacturer’s protocol. Absorbance was measured at 450 nm using an EPOCH plate reader (BioTek, Winooski, VT, USA). The detection limits of ELISA kits were 0.052 ng/mL for lactoferrin and 12 pg/mL for CHI3L1, according to the manufacturer’s information.

### 4.6. Statistical Analysis

Statistical analysis was performed using Statistica v.13 software (TIBCO Software Inc., Palo Alto, CA, USA). Data are presented as the arithmetic mean ± SD. The Shapiro–Wilk test (*α* = 0.05) was used to verify the normality of data distribution. For multiple group comparisons of normally distributed data, one-way ANOVA followed by Tukey’s post hoc test was employed, while the Mann–Whitney U test was used for data with a non-normal distribution. Correlation analysis was conducted using Spearman’s rank correlation coefficient (*r_s_*), and the association between categorical variables was assessed with chi-square (χ^2^) test. Quantitative colocalization analysis was performed using Fiji (ImageJ) version 1.54p with the JaCoP (Just Another Colocalization Plugin) software, which allowed for the calculation of Pearson’s correlation coefficient (*r*), and thresholded Manders’ overlap coefficients (*M*_1_ and *M*_2_).

## Figures and Tables

**Table 1:** Demographic and clinical characteristics of the study groups.

	Untreated MS	DMF-treated MS	Healthy Controls	*p*–Value
*n*	29	23	21	-
age (years, mean ± SD)	36 ± 9	39 ± 7	34 ± 12	*p* > 0.05 ^a^
sex, female/male (*n*)	18/11	16/7	12/9	*p* > 0.05 ^b^
EDSS (mean ± SD)	2.02 ± 1.17	2.02 ± 1.11	-	*p* > 0.05 ^c^
time on DMF (years, mean ± SD)	-	4 ± 2	-	-
previous DMD therapy	-	none	-	-

**Notes:** ^a^ One-way ANOVA followed by Tukey’s post hoc test; ^b^ Chi-square (χ^2^) test; ^c^ Mann–Whitney U test. **Abbreviations:** DMD, disease–modifying drug; DMF, dimethyl fumarate; EDSS, expanded disability status scale; MS, multiple sclerosis; SD, standard deviation.

## Data Availability

The original contributions presented in this study are included in the article. Further inquiries can be directed to the corresponding author(s).

## Abbreviations

Abbreviations used in this manuscript:

BBB	Blood–brain barrier
BSA	Bovine serum albumin
CHI3L1	Chitinase-3-like protein 1
CIS	Clinically isolated syndrome
CLP	Chitinase-like protein
CNS	Central nervous system
CSF	Cerebrospinal fluid
DAPI	4′,6-diamidino-2-phenylindole
DMF	Dimethyl fumarate
DPBS	Dulbecco’s phosphate-buffered saline
EAE	Experimental autoimmune encephalomyelitis
EBV	Epstein–Barr virus
EDSS	Expanded disability status scale
EDTA	Ethylenediaminetetraacetic acid
ELISA	Enzyme-linked immunosorbent assay
GH18	Glycosyl hydrolase family 18
HC	Healthy controls
HL-60	Human promyelocytic leukemia cell line
KHCO_3_	Potassium bicarbonate
MFI	Mean fluorescence intensity
MDM	Disease-modifying drug
MMP-9	Matrix metalloproteinase 9
MPO	Myeloperoxidase
MRI	Magnetic resonance imaging
MS	Multiple sclerosis
NDS	Normal donkey serum
NET	Neutrophil extracellular trap
NH_4_Cl	Ammonium chloride
NK	Natural killer
Nrf2	Nuclear factor erythroid 2-related factor 2
PBMCs	Peripheral blood mononuclear cells
PBS	Phosphate-buffered saline
px	pixel
RBCs	Red blood cells
RRMS	Relapsing-remitting multiple sclerosis
RT	Room temperature
TNFα	Tumor necrosis factor alpha
